# Rational development of multicomponent mRNA vaccine candidates against mpox

**DOI:** 10.1080/22221751.2023.2192815

**Published:** 2023-03-31

**Authors:** Rong-Rong Zhang, Zheng-Jian Wang, Yi-Long Zhu, Wei Tang, Chao Zhou, Suo-Qun Zhao, Mei Wu, Tao Ming, Yong-Qiang Deng, Qi Chen, Ning-Yi Jin, Qing Ye, Xiao Li, Cheng-Feng Qin

**Affiliations:** a State Key Laboratory of Pathogen and Biosecurity, Beijing Institute of Microbiology and Epidemiology, Beijing, People’s Republic of China; b Changchun Veterinary Research Institute, Chinese Academy of Agricultural Sciences, Changchun, People’s Republic of China; c Academicians Workstation of Jilin Province, Changchun University of Chinese Medicine, Changchun, People’s Republic of China; d Research Unit of Discovery and Tracing of Natural Focus Diseases, Chinese Academy of Medical Sciences, Beijing, People’s Republic of China

**Keywords:** Mpox virus, mRNA vaccine, protective antigen, multicomponent, mouse model

## Abstract

The re-emerging mpox (formerly monkeypox) virus (MPXV), a member of *Orthopoxvirus* genus together with variola virus (VARV) and vaccinia virus (VACV), has led to public health emergency of international concern since July 2022. Inspired by the unprecedent success of coronavirus disease 2019 (COVID-19) mRNA vaccines, the development of a safe and effective mRNA vaccine against MPXV is of high priority. Based on our established lipid nanoparticle (LNP)-encapsulated mRNA vaccine platform, we rationally constructed and prepared a panel of multicomponent MPXV vaccine candidates encoding different combinations of viral antigens including M1R, E8L, A29L, A35R, and B6R. *In vitro* and *in vivo* characterization demonstrated that two immunizations of all mRNA vaccine candidates elicit a robust antibody response as well as antigen-specific Th1-biased cellular response in mice. Importantly, the penta- and tetra-component vaccine candidates AR-MPXV5 and AR-MPXV4a showed superior capability of inducing neutralizing antibodies as well as of protecting from VACV challenge in mice. Our study provides critical insights to understand the protection mechanism of MPXV infection and direct evidence supporting further clinical development of these multicomponent mRNA vaccine candidates.

## Introduction

Mpox virus (MPXV) was first isolated from monkeys in 1958, and the first human case of MPXV infection was reported in the 1970s in the Democratic Republic of the Congo [1]. MPXV infection in human causes typical clinical symptoms including fever, headache, lymphadenopathy, myalgia, asthenia, malaise, and skin rash [2]. For a long period of time, MPXV infection cases were documented mainly in Central and African countries with limited human-to-human transmission. Unexpectedly, MPXV expanded its epidemic magnitude and geographic range since May 2022, and finally led to the declaration of Public Health Emergency of International Concern by the World Health Organization (WHO). As of January 2023, more than 83,000 laboratory-confirmed cases of mpox have been reported from 110 countries, including 75 deaths [3]. During this outbreak, some atypical clinical symptoms, characterized by genital and perianal lesions without spread to other sites [4], were also observed. The development of vaccines and antiviral drugs is highly needed for the prevention and treatment of MPXV infection.

MPXV is an enveloped virus with a double-stranded DNA genome which belongs to the *Orthopoxvirus* genus of *Poxviridae* family, together with variola virus (VARV), vaccinia virus (VACV), rabbitpox virus (RPXV) and cowpox virus (CPXV). Most of the protein-coding genes are highly conserved among the members of *Orthopoxvirus* genus [5,6]. Previous studies showed that smallpox vaccine effectively prevents other orthopoxvirus infections in animals, including MPXV, VACV, and RPXV [6–9]. An early study reported that individuals immunized with smallpox vaccine exhibited 85% protection against MPXV infection [10]. Currently, two smallpox vaccines have been conditionally approved by the U.S. Food and Drug Administration (FDA) for the prevention of MPXV infection. The JYNNEOS vaccine is a modified vaccinia virus (Ankara) based live, nonreplicating vaccine [11], and the other one ACAM2000 is a traditional live attenuated vaccine [12]. However, the accessibility as well as the potential safety concerns of the two vaccines are far from the medical demand worldwide. More importantly, a recent study revealed that immunization with JYNNEOS induced relatively low levels of neutralizing antibodies against MPXV [13]. Thus, a safe, effective, and accessible MPXV-specific vaccine is urgent needed to face the ongoing mpox epidemic.

Messenger RNA (mRNA)-based vaccine has been well demonstrated as an effective technology to prevent infectious diseases with advantages including rapid and scalable production, excellent safety profile without nuclear entry, and the ability to effectively induce humoral and cellular immune responses. During the coronavirus disease 2019 (COVID-19) pandemic, mRNA vaccines developed by Moderna and Pfizer/BioNTech have been authorized for use in humans for the first time [14]. Inspired by the great success of COVID-19 mRNA vaccines, the development of MPXV-specific mRNA vaccine represents the most attractive strategy in response to mpox epidemic [15]. However, the immune protection mechanism of MPXV infection remains elusive, and the protective antigens of MPXV remain not fully characterized [16,17]. Like other orthopoxviruses*,* MPXV has two infectious forms: the intracellular mature virion (IMV) and the extracellular enveloped virion (EEV), both are infective and capable of inducing diseases. The ∼200 kb genome of MPXV encodes at least 190 proteins, and more than 30 of them were known as structural proteins [18,19]. Previously, a series of protective antigens of orthopoxvirus were uncovered and utilized in the design of subunit vaccines or DNA vaccines, including several IMV surface proteins (L1R, D8L, and A27L) and EEV surface proteins (A33R, and B5R) of VACV [20-23]. Among these, L1R, D8L, A27L, and B5R contained critical neutralization epitopes and were demonstrated to elicit neutralizing antibodies in animals and human [20,21,24,25]. Also, A33R could enhance the protection conferred by L1R immunization in mouse [26,27]. In addition, antibodies induced by A33R are able to inhibit comet formation by VACV in cells and prevent subsequent infection [21,26]. Accumulated evidence has demonstrated that the combination of multiple antigens from both IMV and EEV is the prerequisite to provide sufficient protection against poxvirus challenge in animals [20–23,28]. Previously, a pentavalent subunit vaccine encoding the five proteins of VACV (L1R, D8L, A27L, A33R, and B5R) possessed a highly improved protection efficacy compared with the quadrivalent formulation (L1R, A27L, A33R, and B5R), with the survival rate increasing from 26% to 66% against lethal VACV challenge in mouse model [21]. Thus, an ideal MPXV mRNA vaccine is supported to encode “tailored combination” of multiple protective antigens from both EEV and IMV of MPXV.

In the present study, we developed a panel of multicomponent MPXV vaccine candidates encoding different combinations of viral antigens including M1R, E8L, A29L, A35R, and B6R. Further experiments clearly demonstrated that two immunizations of all mRNA vaccine candidates elicit a robust antibody response as well as antigen-specific T cell immune response in mice. Importantly, the penta- and tetra-component vaccine candidates showed superior protection efficacy based on a mouse model of VACV infection.

## Materials and methods

### Cells and viruses

BS-C-1 (ATCC, CCL-26) cells were cultured in Minimum Essential Medium (MEM, Procell, China) containing 10% fetal bovine serum (FBS, Gibco, USA) and 1% penicillin–streptomycin (PS, Gibco, USA) with 5% CO_2_. VACV Western Reserve (WR) strain [29] and Tian Tan strain (GenBank accession no. AF095689.1) were grown on BSC-1 cells. FBS used in all experiments was heat-inactivated prior to use. BS-C-1 cells were infected with VACV at a multiplicity of infection (MOI) of 0.5. Cells were harvested at 2 days post infection (dpi), suspended in phosphate buffered solution (PBS, Solarbio, Beijing, China) and lysed by rapidly freezing and thawing. Cell debris was removed by centrifugation at 1200 g for 5 min. Purified stocks were made via centrifugation. Virus titres were determined by standard plaque assay on BS-C-1 cells and virus stocks were stored at −80°C until use.

### mRNA preparation and characterization

All DNA sequences encoding A35R, B6R, M1R, A29L, and E8L were synthesized (Sangon Biotech, China) and cloned into the plasmid ABOP-028 (GENEWIZ, China) [15]. The mRNAs were produced *in vitro* using T7 RNA polymerase-mediated transcription from linearized DNA templates.

BS-C-1 cells were seeded onto 24-well plates with 10^5^ cells per well and transfected with 2 μg of M1R-, E8L-, A35R-, A29L-, or B6R-encoding mRNA using Lipofectamine^TM^ MessengerMAX^TM^ (Thermo Fisher Scientific, USA), respectively. At 24 h after transfection, cells were fixed with acetone–methanol (3/7) at room temperature for 15 min and incubated with anti-MPXV M1R antibody (1:300, AntibodySystem, China), anti-MPXV E8L antibody (1:500, AntibodySystem, China), anti-MPXV A35R antibody (1:300, AntibodySystem, China), or anti-Vaccinia Virus (1:1000, GeneTex, USA, Abcam, Cambridge, UK) antibody at 37°C for 2 h. Cells were then washed with PBS and incubated with Alexa Fluor 488-conjugated anti-mouse secondary antibody (1:200, Thermo Fisher Scientific, USA) for 1 h at 37°C. The cells were subsequently incubated with DAPI (1:5000, Sigma, USA) for 5 min. Images were acquired by the Olympus BX51 microscope under the control of DP 72 software.

### Lipid-nanoparticle (LNP) encapsulation of mRNA

LNP formulations were prepared using the same procedure as described previously [15]. Briefly, lipids including ionizable lipids, 1-,2-distearoyl-sn-glycero-3-phosphocholine (DSPC), cholesterol and Polyethylene Glycol (PEG)-lipid (molar ratios of 50:10:38.5:1.5) was dissolved in ethanol. Then, the lipid mixtures were combined with 20 mM citrate buffer (pH 4.0) containing mRNA at a ratio of 1:2 through a T-mixer. The formulation was diafiltrated in 10 x volume of PBS (pH 7.4) through a tangential flow filtration membrane (100 kD), and filtrated using a 0.22 mm filter. Final LNP formulations were transferred to a new tube and stored at 2–8°C until use. The particle size, distribution, RNA concentration, and encapsulation were tested. The mRNA vaccine candidates AR-MPXV5, AR-MPXV4a, AR-MPXV4b, and AR-MPXV3 were developed by mixing antigen-encoding mRNA-LNPs as different combinations under the same mRNA vaccine platform. Empty LNPs were utilized as placebo.

### Vaccination and mouse challenge experiment

All animal procedures were reviewed and approved by the Animal Experiment Committee of Laboratory Animal Center, Academy of Military Medical Sciences (AMMS) (approval number: IACUC-DWZX-2022-055). 5-week-old female BALB/c mice were immunized intramuscularly with AR-MPXV5, AR-MPXV4a, AR-MPXV4b, or AR-MPXV3 (containing 5 μg of each antigen-encoded mRNA), respectively, and boosted with the same dose after 3 weeks. Serum samples were collected at 14, 21, 28, and 35 days after initial immunization.

Immunized mice were challenged intranasally with 10^6^ PFU of VACV (strain Tian Tan, GenBank accession no. AF095689.1) 91 days post-initial immunization. Weight loss was monitored for 20 days; some infected animals (AR-MPXV5, *n* = 4; AR-MPXV4a, *n* = 3; AR-MPXV4b, *n* = 4; AR-MPXV3, *n* = 4; placebo, *n* = 4) were sacrificed 8 days post-challenge for tissue harvest and virological analyses.

### Enzyme-linked immunosorbent assay (ELISA)

ELISA was used to determine the IgG antibody titres in the sera samples. Briefly, 96-well plates were coated overnight at 4°C with VACV WR strain (10^5^ PFU/well) diluted with 10× coating buffer (Solarbio, China). Serum samples were heated at 56°C for 30 min before use. Inactivated serum samples were two-fold serially diluted, added to blocked 96-well plates, and incubated at 37°C for 2 h. Then, secondary antibody (HRP-conjugated reagent, 1:10,000) was added to each well of the plates and incubated for 1 h at 37°C. Soluble TMB (Cwbio, China) was used and incubated for 15 min at room temperature. The reaction was stopped by the addition of stop solution (Solarbio, China) and the absorbance (450 nm) was read using Synergy H1 hybrid multimode microplate reader (BioTek, USA). The endpoint titres were defined as the highest reciprocal serum dilution that yielded an absorbance >2-fold over background values.

### Plaque reduction neutralization test (PRNT)

The neutralizing antibody titres in sera samples were determined by 50% plaque reduction neutralization test (PRNT_50_). Twofold serial dilutions of serum samples were inactivated at 56°C for 30 min, and incubated for 90 min at 37°C with an equal volume of VACV WR strain containing ∼250 PFU of virus per mL. Samples were then applied to BS-C-1 cell culture monolayers in 12 well plates and incubated for 90 min at 37°C. Cells were overlaid with 0.5% methylcellulose in Dulbecco’s minimal essential medium (DMEM, Gibco, USA) with 2.5% inactivated FBS, incubated for 2 days at 37°C in 5% CO_2_. Viral titres were then determined using the plaque assay described above. The PRNT_50_ titres were calculated by the method of Spearman–Karber.

### Flow cytometry analyses

Intracellular cytokine staining was used to measure MPXV antigen-specific T cells in immunized mice. Briefly, a total of 1,000,000 mouse splenocytes were co-stimulated with overlapping antigen peptide pools including M1R, E8L, A29L, A35R, and B6R (1.5μg/ml of each peptide, GenScript, China) for 1 h at 37°C in 5% CO_2_, respectively, together with 1 µg/ml anti-mouse CD28 antibody (Biolegend, USA) and CD49d antibody (Biolegend, USA). Then, Protein Transport Inhibitor (1:1000, BD Biosciences, USA) was added to splenocytes and incubated for 8 h at 37°C in 5% CO_2_. Cells were collected and washed twice with PBS containing 2% FBS, blocked with anti-CD16/CD32 antibody (1:200, Biolegend, USA) and stained with Zombie Aqua™ Fixable Viability Kit (1:200, Biolegend, USA), fluorescently conjugated antibodies to CD3 (1:200, BV421, Biolegend, USA), CD4 (1:200, FITC, BD Biosciences, USA), and CD8 (1:200, APC/Cyanine7, Biolegend, USA) for 30 min at 4°C in the dark. Subsequently, splenocytes were washed twice with PBS containing 2% FBS, fixed and permeabilized using the Cytofix/Cytoperm kit (BD Biosciences, USA), and then stained with fluorescently conjugated antibodies to interferon-γ (IFN-γ) (1:200, PE, Biolegend, USA), interleukin-2 (IL-2) (1:200, PE-Cy™7, Biolegend, USA), interleukin-4 (IL-4) (1:200, APC, Biolegend, USA), and tumour necrosis factor alpha (TNF-α) (1:200, PerCP/Cyanine5.5, Biolegend, USA) for 30 min at 4°C in the dark. Finally, splenocytes were washed twice, resuspended with PBS containing 2% FBS, detected on FACSVerse Flow Cytometer (BD Biosciences, USA) and analysed with FlowJo software.

### Measurement of infectious virus particles and viral genome

Tissue homogenates were clarified by centrifugation at 8,000 rpm for 10 min. Infectious virus particles in the supernatants were detected using standard plaque assay on BS-C-1 cells. Viral nucleic acids were extracted by using the Viral RNA/DNA Extraction Kits (TIANLONG, China) according to the manufacturer’s protocol. DNA quantification was performed by qPCR using Probe qPCR Mix (Takara, Japan) with the following primers and probes: VACV-F (5′- GGCAATGGATTCAGGGATATAC-3′), VACV-R (5′- ATTTATGAATAATCCGCCAGTTAC), and VACV-P (5′- CAATGTGTCCGCTGTTTCCGTTAATAAT-3′). PCR was conducted in a LightCycler® 480 Instrument (Roche Diagnostics Ltd, Switzerland).

### Statistical analyses

Statistical analyses were carried out using Prism software (Graphpad Prism 7.0). Data are presented as mean ± SEM in all experiments. Detailed information of statistical analyses was stated in the relevant figure legends.

## Results

### Rational design and characterization of multicomponent mRNA vaccine candidates against mpox

**Figure 1.**
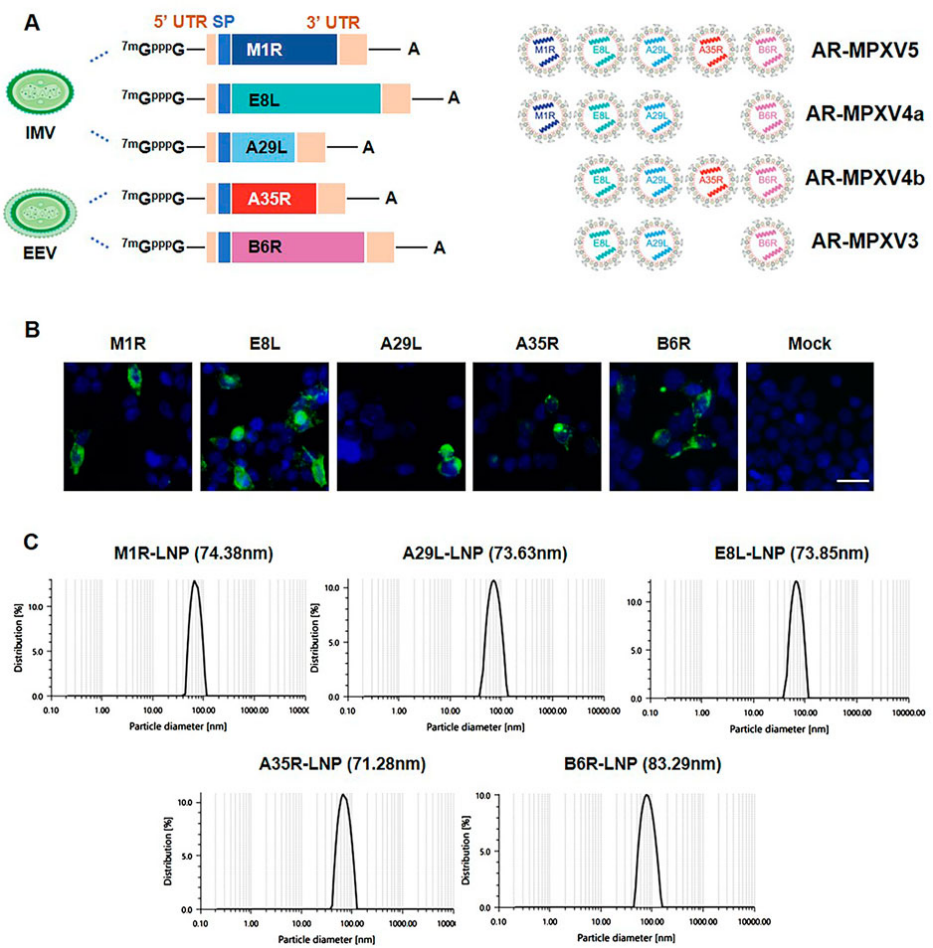
Design and characterization of MPXV mRNA vaccine candidate encoding multiple antigens. (A) Construction and encapsulation of mRNA-LNPs encoding multiple proteins of MPXV. (B) MPXV protein expression in HEK293T cells. Cells were transfected with antigen-encoded mRNAs and detected at 24 h post transfection by indirect immunofluorescence staining. Scar bar, 50 μm. (C) Representative size distribution graph of A35R-LNP, B6R-LNP, M1R-LNP, A29L-LNP and E8L-LNP.

To rationally design and develop a MPXV mRNA vaccine candidate, we first constructed five individual mRNA that encodes the five known protective antigens (Figure 1(A) and Supplementary Figure S1), including the IMV-derived antigens M1R, E8L, and A29L, and EEV-derived antigens A35R and B6R, based on the sequence of a newly identified MPXV isolate MPXV_USA_2022_MA001 (GenBank accession no. ON563414.2). The *in vitro* expression of each mRNA was confirmed by indirect immunofluorescence assay in HEK293 T cells, respectively (Figure 1(B)). Then, the antigen-encoded mRNAs were separately processed into LNP formulations as previously described [15]. As shown in Figure 1(C), dynamic light-scattering analyses showed that all of the final mRNA-LNP formulations exhibited similar average particle sizes ranging from 71.28 to 83.29 nm.

### Multicomponent mRNA vaccine elicits a robust humoral immune response in mice

**Figure 2.**
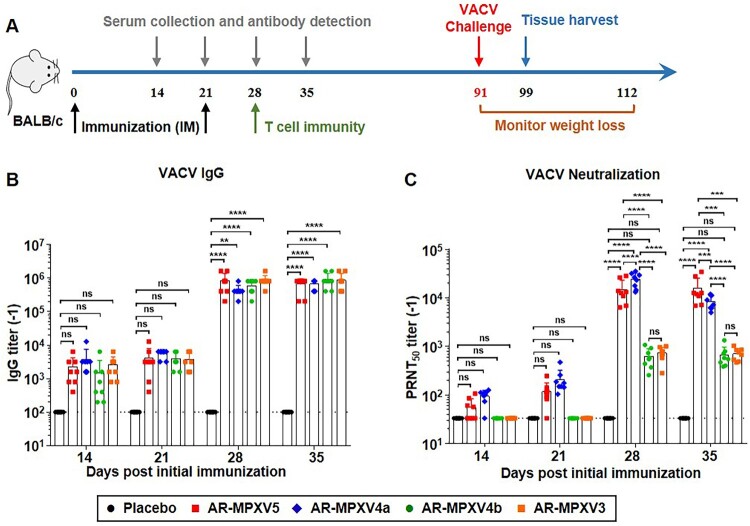
Multicomponent mRNA vaccine induces a robust antibody response in mice. Groups of BALB/c mice were immunized with mRNA vaccine or placebo and boosted with an equal dose 3 weeks later. Sera sample were collected at indicated times post immunization. (A) Schematic diagram of immunization and challenge experiment. (B) VACV specific IgG antibody titres were determined by ELISA. (C) Neutralizing antibody levels against VACV were determined by PRNT assay. Data are shown as mean ± SEM. Significance was analysed by two-way ANOVA with multiple comparisons tests (ns, not significant, **p < 0.01, ***p < 0.001, ****p < 0.0001).

To determine the most potent combinations of different mRNA-LNP formulations, four candidate formulations containing an equal amount of each antigen-encoded mRNA were prepared and named as AR-MPXV5 (M1R + E8L + A29L + A35R + B6R), AR-MPXV4a (M1R + E8L + A29L + B6R), AR-MPXV4b (E8L + A29L + A35R + B6R) and AR-MPXV3 (E8L + A29L + B6R), respectively (Figure 1(A)). Each of the combinations contained at least one antigen from IMV and EEV, respectively. Then, all combinations were subjected to standard immunogenicity assay in BALB/c mice. Groups of 5-week-old mice were intramuscularly immunized with AR-MPXV5, AR-MPXV4a, AR-MPXV4b, or AR-MPXV3, respectively, and boosted with the same dose after 3 weeks (Figure 2(A)). Empty LNPs were used as Placebo. At 14, 21, 28, and 35 days post-initial immunization, sera samples were collected and IgG antibody responses against VACV [29] were determined by ELISA. Clearly, a single immunization with all vaccine candidates induced robust IgG antibody response and a second immunization resulted in a rapid improvement of IgG antibody levels (Figure 2(B)). There was no significant difference among the four immunized groups. Standard PRNT was then performed by using the VACV WR strain [29]. Remarkably, high levels of neutralizing antibodies were detected in AR-MPXV5- and AR-MPXV4a-immunized mice, while the neutralizing antibody was undetectable in AR-MPXV4b- and AR-MPXV3-immunized mice 14 and 21 days post-initial immunization (Figure 2(C)). After the second immunization, neutralizing antibodies of all immunized groups were significantly elevated and the PRNT_50_ titres reached ∼1:13,383 (AR-MPXV5), ∼1:8156 (AR-MPXV4a), ∼1:606 (AR-MPXV4b), and ∼1:683.9 (AR-MPXV3) 35 days post-initial immunization. Neutralizing antibody titres of the AR-MPXV5-, AR-MPXV4a-immunized groups were significantly higher than that of the AR-MPXV4b-, AR-MPXV3-immunized groups (Figure 2(C) and Supplementary Figure S2).

### Multicomponent mRNA vaccine effectively elicits a MPXV antigen-specific T cell immune response

**Figure 3.**
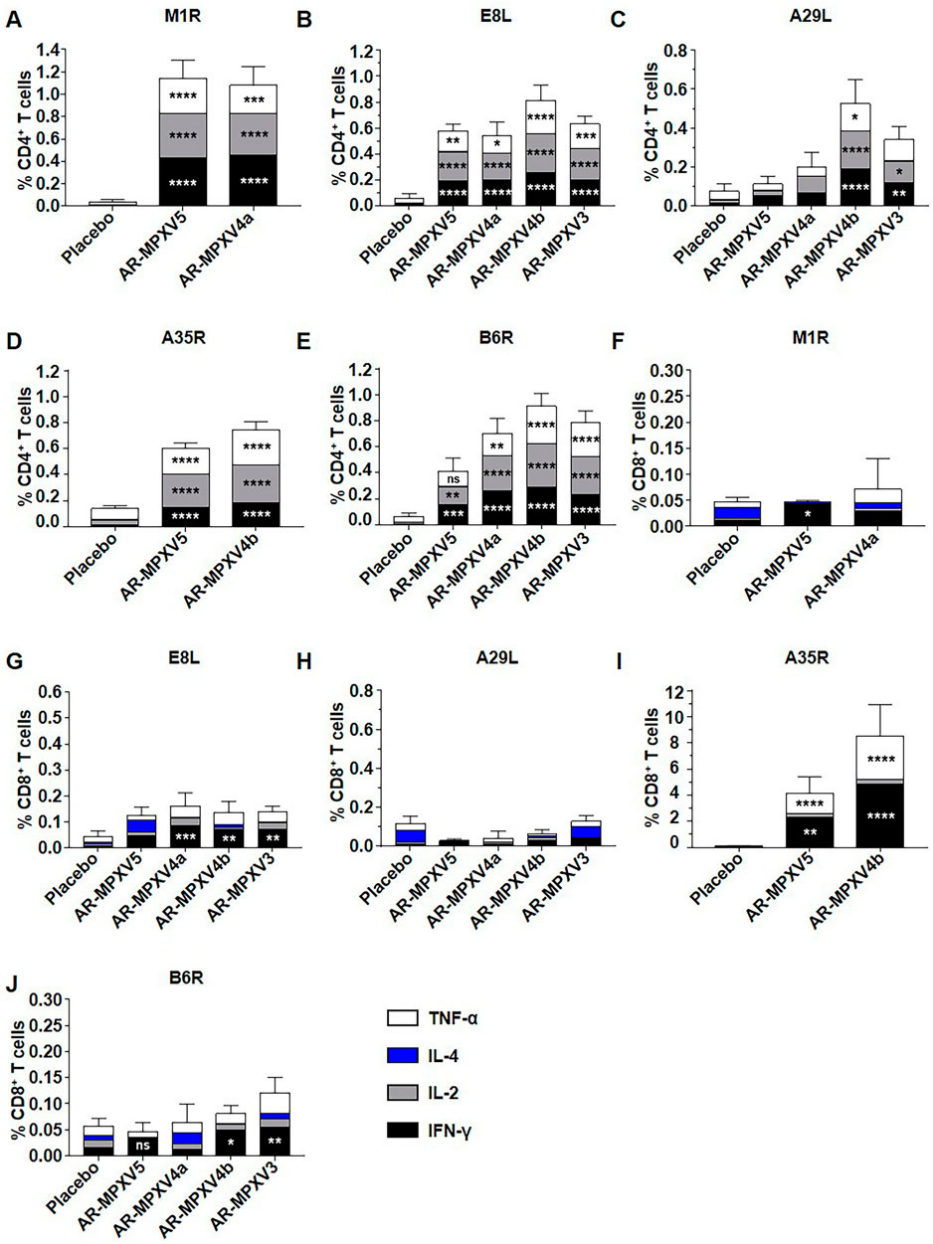
Antigen-specific CD4^+^ and CD8^+^ T cell responses following immunization. BALB/c mice were immunized i.m. with two doses of multicomponent mRNA vaccine. (A–J) Flow cytometry assay for IFN-γ, IL-2, IL-4 and TNF-α in splenocytes. Spleen was harvested and stimulated with MPXV A35R (A, F), B6R (B, G), M1R (C, H), A29L (D, I) and E8L (E, J) peptide pools 7 days after two immunizations. Data are shown as mean ± SEM. Significance was analysed by two-way ANOVA with multiple comparisons tests (ns, not significant, *p < 0.05, **p < 0.01, ***p < 0.001, ****p < 0.0001).

Then, MPXV antigen-specific T cell immune response was evaluated 7 days after the second immunization. We characterized the immune phenotype of the resulting cellular response for IFN-γ, IL-2, IL-4, and TNF-α stimulated by the respective antigen peptide pools, including M1R, E8L, A29L, A35R, and B6R. Compared with the placebo, all of the four multicomponent MPXV mRNA vaccine candidates elicited antigen-specific CD4^+^ T cells expressing type 1 (Th1) immune response cytokines (IFN-γ, IL-2, and TNF-α) in the spleen (Figure 3(A–E)). And there was no significant difference in the induction of IL-4 between the vaccine- and placebo-immunized groups (Figure 3(A–E)). Apparently, two immunizations of mRNA vaccine successfully induced a robust Th1-biased, antigen-specific cellular immune response. Meanwhile, we detected obvious M1R-, E8L-, A35R-, and B6R-specific CD8^+^ T cell responses in the vaccine immunized groups, especially the vastly significant A35R-specific CD8^+^ T cells expressing IFN-γ and TNF-α in the AR-MPXV5- and AR-MPXV4b-immunized mice (Figure 3(F–G, I–J)). However, the A29L-specific CD8^+^ T cell response was not detected in all of the immunized groups (Figure 3(H)).

### Multicomponent mRNA vaccine protects mice from VACV challenge

**Figure 4.**
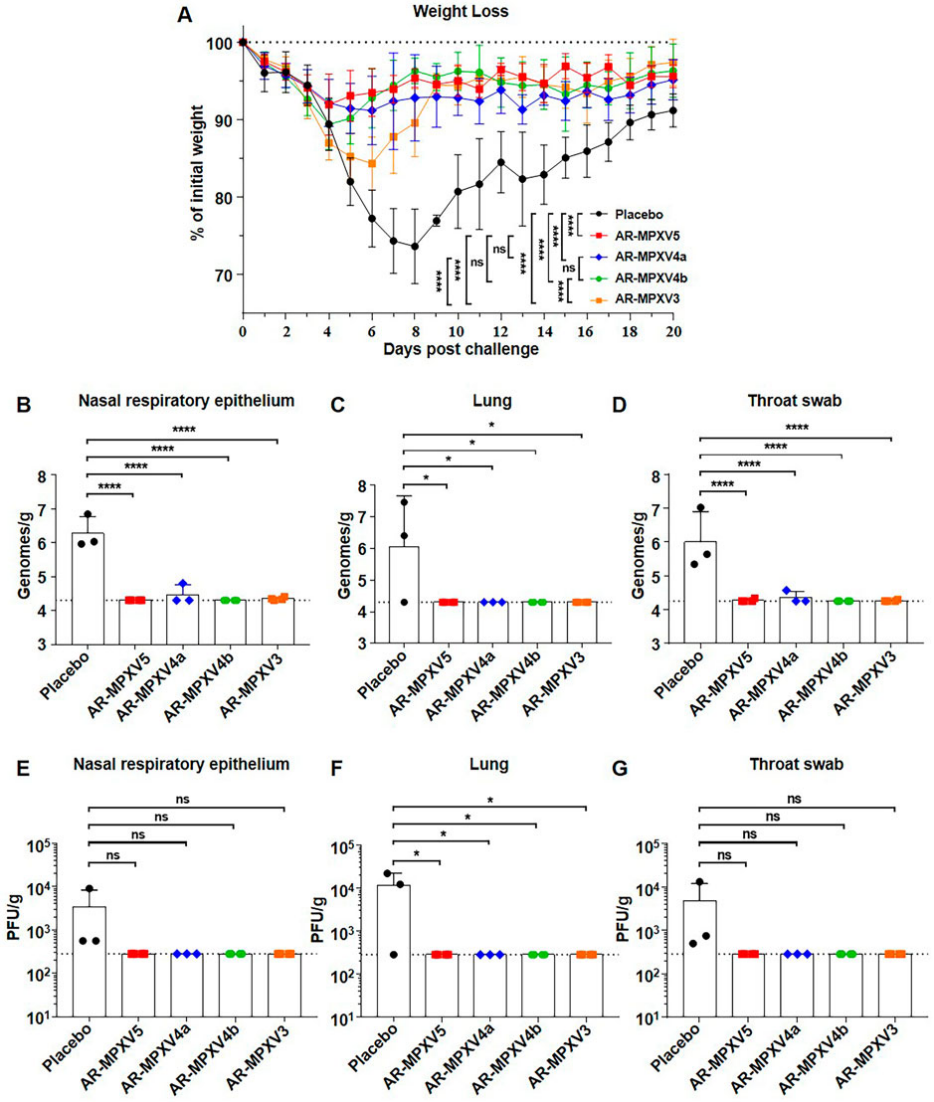
Multicomponent mRNA vaccine protects mice from VACV challenge. (A) Groups of mice immunized with mRNA vaccine or placebo were intranasally challenged with 10^6^ PFU of VACV. Weight changes were monitored for 20 days post infection. (B–D) Viral genome copies in nasal respiratory epithelium (B), lung (C) and throat swab (D) were determined by qPCR. (E–G) VACV titres in nasal respiratory epithelium (E), lung (F) and throat swab (G) were measured using standard plaque assay in BSC-1 cells. Data are shown as mean ± SEM. Significance was analysed by one-way ANOVA or two-way ANOVA with multiple comparisons tests (ns, not significant, ns, not significant, *p < 0.05, ****p < 0.0001).

Finally, VACV (Tian Tan strain, GenBank accession no. AF095689.1) was used to evaluate the protective efficacy of the multicomponent mRNA vaccines in mice. The Tian Tan strain shares a 98% nucleotide sequence identity with MPXV_USA_2022_MA001 (GenBank accession no. ON563414.2) and both the nucleotide and amino acid sequence of these antigens (L1R, D8L, A27L, A33R, and B5R) are highly homologous to the ones (M1R, E8L, A29L, A35R, and B6R) we used (Supplementary Table S1). All of the immunized mice were challenged intranasally with 10^6^ PFU of VACV 91 days post-initial immunization (Figure 2(A)). Weight changes were recorded for 20 days post-challenge. As expected, the placebo-immunized mice showed a significant weight loss of up to 26.3% at 8 days upon VACV challenge (Figure 4(A)). AR-MPXV3-immunized mice experienced obvious weight loss as much as 15.7% within 6 days after infection, while AR-MPXV4b-immunized mice lost 10.7% of their initial weight within 4 days. Notably, only a transient and slight weight loss of less than 10% (8.1%, 8.8%) was observed in the AR-MPXV5- or AR-MPXV4a-immunized group. Meanwhile, viral load was detected in the nasal respiratory epithelium, lung, and throat swob 8 days post-challenge by qPCR (Figure 4(B–D)) and standard plaque assay (Figure 4(E–G)). As expected, both viral DNA and infectious virus particles were detected in the placebo-immunized group, while only marginal viral DNA genome were detected in the AR-MPXV5-, AR-MPXV4a-, and AR-MPXV3-immunized groups. No viral DNA or infectious particles were detected in the AR-MPXV4b immunized groups. The results indicated that all of the vaccine candidates protected mice from losing weight after a high-dose of VACV infection. The animals immunized with AR-MPXV5 or AR-MPXV4a were most well-protected as producing high levels of antibodies as well as losing less weight than the other groups after challenge.

## Discussion

In this study, we developed a panel of multicomponent mRNA vaccine candidates against MPXV as various combinations of mRNA-LNPs encoding several IMV surface proteins (M1R, E8L, and A29L) and EEV surface proteins (A35R and B6R). The results showed that penta- and tetra-component vaccines AR-MPXV5 and AR-MPXV4a successfully induced a potent neutralizing antibody response and exhibited a robust protection against VACV infection in a mouse model. On the contrary, tetra-component vaccine candidate AR-MPXV4b and tricomponent vaccine candidate AR-MPXV3 lacking of M1R resulted in a significant reduction of vaccine-induced neutralizing antibodies and more weight loss after VACV challenge (Figure 2(C–D)), emphasizing the indispensable component of M1R. A previous study reported a subunit smallpox vaccine consisting of four proteins (L1R, A27L, A33R, and B5R) conferred a complete protection from VACV challenge in a mouse model; in contrast, mice immunized with three proteins (A27L, A33R, and B5R) lacking of L1R experienced sustained weight loss and more than 80% of infected animals died after lethal challenge [30]. These results also highlighted the significant protection efficacy of L1R as a protective immunogen (highly homologous with M1R of MPXV). Obviously, the penta- and tetra-component vaccine candidates AR-MPXV5 and AR-MPXV4a exhibited superior capability of inducing neutralizing antibodies as well as of protecting from VACV challenge in mice.

Previous investigation showed that B cells and immunoglobulins are key elements of humoral immunity and are essential for the protection against MPXV infection [1,31,32]. In a previous study of smallpox DNA vaccine, we noticed that 10 μg of DNA vaccine expressing L1R, D8L, A27L, A33R, and B5R antigens induced neutralizing antibodies (∼1:1000) against VACV [21]. 10^8^ PFU of a highly attenuated candidate smallpox vaccine (modified vaccinia virus Ankara, MVA), by contrast, induced robust neutralizing antibodies against VACV with a PRNT_50_ titre of 1:12,130 after two immunizations [7]. In our study, the penta- and tetra-component mRNA vaccine candidates AR-MPXV5 and AR-MPXV4a successfully induced extremely potent neutralizing antibodies against VACV 28 days post-initial immunization (Figure 2(C)). The PRNT_50_ titres substantially increased to 1:13,092 and 1:22,770, respectively. Besides, the neutralizing activity of AR-MPXV5- and AR-MPXV4a-immunized sera against different MPXV strains will be further determined in the future. On the other hand, monocytes and macrophages have been reported as the initial targets of MPXV infection [33–35]. The activation of CD8^+^ T cells was verified to kill orthopoxvirus-infected macrophages and monocytes to inhibit virus spreading [1,36], as well as protect against lethal virus challenge in animals [37,38]. In our study, significant antigen-specific CD8^+^ T cell responses were elicited by the multicomponent mRNA vaccines, especially the A35R-specific CD8^+^ T cells in the AR-MPXV5 and AR-MPXV4b immunized groups. Also, our multicomponent mRNA vaccines induced a Th1-prone CD4^+^ T cell immune response to different antigens (Figure 3(A–E)). Similarly, significant CD8^+^ T cells expressing IFN-γ were detected in MVA-immunized mice [7] and a Th1-biased antigen-specific cellular immune response was detected in cynomolgus macaques after three immunizations with multivalent smallpox DNA vaccine [28].

MPXV can be classified into two distinct clades: the Central African clade and West African clade. The Central African clade is generally considered to be more virulent [39]. The mpox outbreak in 2022 belongs to lineage B.1 of the West African clade (clade 3) and originates from a single origin associated with an outbreak in 2018–2019 [39]. About 50 single-nucleotide polymorphisms (SNPs) have been observed during the outbreak, suggesting a higher rate of variation than expected [39,40]. In addition, large deletions, insertions, and genomic rearrangements have also been reported recently in the circulating MPXV strain [41]. The coding sequences of our MPXV vaccine candidate were derived from a contemporary MPXV strain that belongs to clade 3, and the corresponding antigens chosen are highly conserved between the Central African and West African clade. Whatever, the cross neutralization and protection of our current vaccine candidates among different MPXV clades or strains remains to be determined.

To date, MPXV-challenged rodent and non-human primate models have been successfully established and used to demonstrate the protective capacity of MPXV vaccine [6,28,31,42–45]. For instance, DNA vaccine and viral vector-based vaccine have been confirmed to provide protection against lethal MPXV infection in rhesus macaques and 129 *stat1^-/-^* mice [6,43]. Our data from BALB/c mice showed that MPXV mRNA vaccine candidates could provide effective protection from losing weight after a high-dose of VACV infection. It’s worthwhile to further test the protection efficacy in other animal models especially the non-human primate challenge model with the circulating MPXV strain before clinical trials.

Overall, we have rationally developed and evaluated two unique penta- and tetra-component mRNA vaccine candidates against mpox, and the promising efficacy data from mice as well as the maturity of mRNA vaccine platform support further development. Meanwhile, our study provides critical clues about the distinct role of different protective antigens of MPXV that will deepen our understanding of the immune protection mechanism of MPXV infection.

## Author contributions

C.F.Q., Q.Y., and R.R.Z. conceived and designed the project. R.R.Z., Z.J.W., Y.L.Z., W.T., and C.Z. performed experiments. Q.Y., R.R.Z., Z.J.W., W.T., C.Z., S.Q.Z., M.W., T.M., Y.Q.D., Q.C., and N.Y.J. analysed data. C.F.Q., X.L., Q.Y., and R.R.Z. wrote and finalized the manuscript. All authors read and approved the manuscript.

## Supplementary Material

Supplemental Material
