# Supplementary material for: Rational development of multicomponent mRNA vaccine candidates against mpox
Source: Emerg Microbes Infect. 2023 Mar 31;12(1):2192815. doi: 10.1080/22221751.2023.2192815 (PMC10071941; doi:10.1080/22221751.2023.2192815)
Supplement: Supplemental Material [file TEMI_A_2192815_SM8825.docx]

**Supplementary Materials**

**Figure S1.** Amino acid sequence of MPXV antigens used in this study.

**Figure S2.** Neutralizing activity of mRNA vaccine immunized sera samples 35 days post-initial immunization in mice.

**Table S1.** The homology of the five antigens of MPXV_USA_2022_MA001 with VACV (Tian Tan strain).

|  | Sequence  Identity | MPXV (MPXV_  USA_2022_MA001) | VACV (Tian Tan strain) |
| --- | --- | --- | --- |
| Nucleotide | 98.27% | M1R | L1R |
|  | 95.85% | E8L | D8L |
|  | 97.00% | A29L | A27L |
|  | 97.96% | A35R | A33R |
|  | 97.06% | B6R | B5R |
| Amino Acid | 98.40% | M1R | L1R |
|  | 94.08% | E8L | D8L |
|  | 94.55% | A29L | A27L |
|  | 96.11% | A35R | A33R |
|  | 97.16% | B6R | B5R |
